# Understanding how foods and enteral feedings influence the gut microbiome

**DOI:** 10.1002/ncp.11285

**Published:** 2025-03-06

**Authors:** Gail A. M. Cresci

**Affiliations:** ^1^ Department of Gastroenterology Hepatology, and Nutrition, Digestive Disease Institute, Cleveland Clinic Cleveland Ohio USA; ^2^ Department of Inflammation and Immunity Lerner Research Institute, Cleveland Clinic Cleveland Ohio USA

**Keywords:** adult, enteral formulas, fiber, microbiome, nutrition

## Abstract

The gut microbiome supports both gut and overall health. Diet is known to be one of the driving factors that influences the gut microbiome. The foods we eat, the dietary and nondietary components they contain, various food consumption patterns, and the ratio of nutrients consumed have been shown to impact gut microbiome composition and function. Studies indicate that many acute and chronic diseases are associated with alterations to the gut microbiome. There are many patients who rely on enteral tube feeding for their nutrition support. More recently, enteral tube feeding formulations of “real food” have become commercially available. However, little is known about how enteral tube feeding impacts the gut microbiome in patients requiring this specialized form of nutrition therapy. This review summarizes the existing evidence regarding the food sources of commonly consumed macronutrients and their impact on the gut microbiome. Also presented is what is known regarding “standard” and real food enteral formulations on the gut microbiome. Existing evidence is suggestive that real food enteral formulations positively impact the gut microbiome. Still, more research is needed on ready‐to‐feed formulations, particularly in patients with various clinical conditions, and how gut microbiome modulation impacts clinical outcomes.

## INTRODUCTION

**Figure 1:**
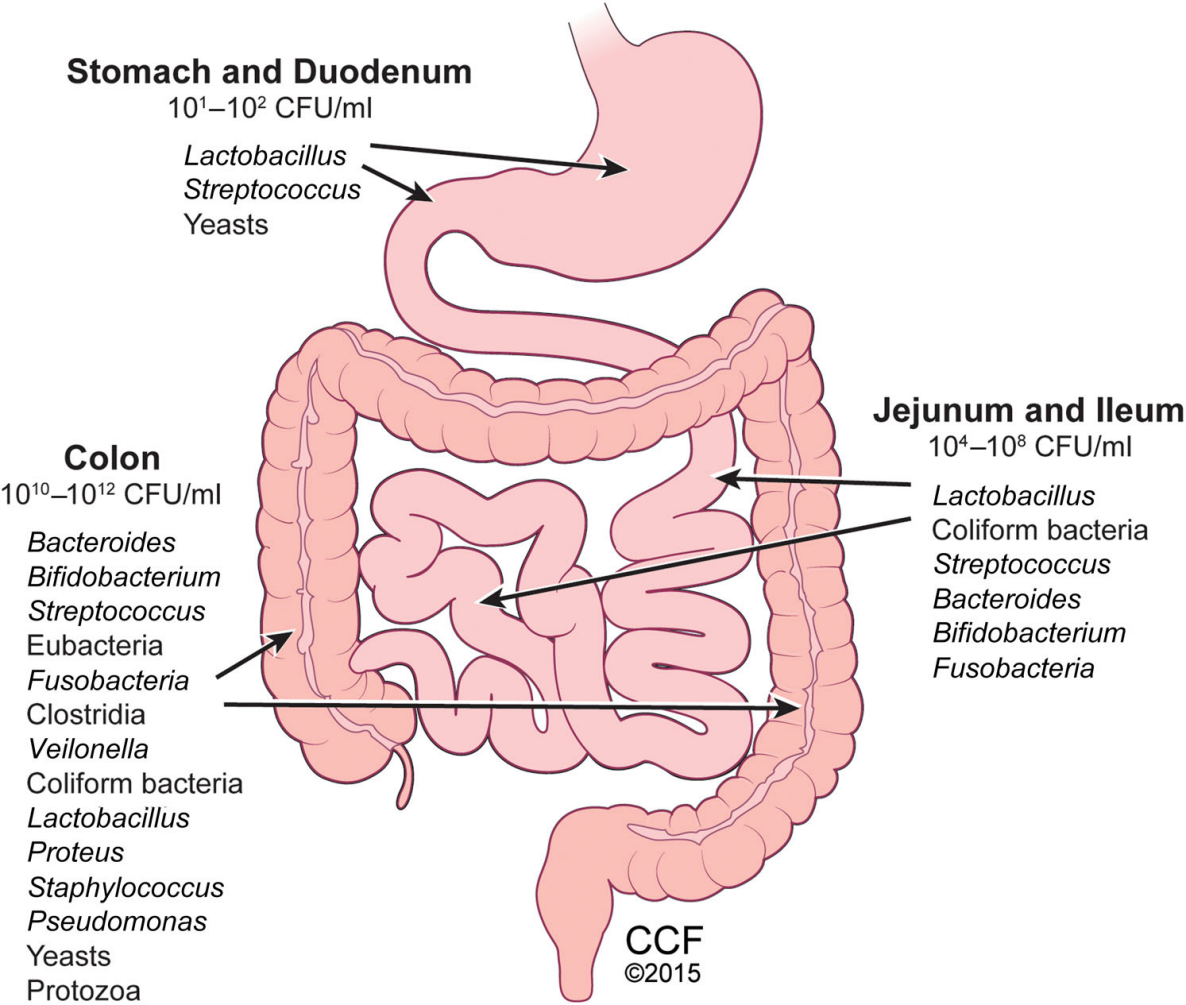
The human gut microbiome. Trillions of microbes comprise the gut microbiome, including bacteria, yeast/fungi, viruses, and protozoa. Although microbes reside throughout the gut, they are found at the highest density in the colon. CCF, Cleveland Clinic Foundation; CFU, colony‐forming unit. Reprinted with the permission of the Cleveland Clinic Center for Medical Art & Photography copyright 2015.

The gut microbiota forms a complex ecosystem composed of trillions of microbes including bacteria, fungi/yeasts, viruses, protozoa, archaea, parasites, and phages, which are located predominantly in the distal gut (Figure 1). We know most about the gut bacteria. The adult human gut microbiota, often termed the “hidden organ,” is composed of six major bacterial phyla: Firmicutes (synonym [syn]: Bacillota), Bacteroidetes (syn: Bacteroidota), Proteobacteria (syn: Pseudomonadota), Actinobacteria (syn: Actinomycetota), Fusobacteria (syn: Fusobacteriota), and Verrucomicrobiota, with Firmicutes and Bacteroidetes being the major phyla representing 90% of the gut microbiota.^1^ The Firmicutes phylum is comprised of >250 different Gram‐positive genera, such as *Lactobacillus, Bacillus, Enterococcus*, and *Ruminococcus*, as well as *Clostridia*, which comprise 95% of the Firmicutes phylum. Some genera in the Firmicutes phylum can produce beneficial metabolites such as short‐chain fatty acids (SCFAs), which can help with gut inflammation, energy production, and gut barrier integrity, and other genera (eg, *Bacillus stercoris*) that have antibacterial properties against pathogenic bacteria.^2^ The Bacteroidetes phylum contains many Gram‐negative bacteria of genera *Bacteroides, Prevotella*, and *Allistipes*. Bacteroidetes are typically known to be friendly bacteria that ferment polysaccharides to produce SCFAs, convert primary conjugated to deconjugated bile acids (*B. fragilis*), or provide colonization resistance to pathogenic bacteria such as *Clostridioides difficile*.^2^ However, an imbalance in gut microbiota can result in certain genera of Bacteroidetes (eg, *Bacteroides*) to shift and possess negative effects and associate with harmful infections.^3^ The Proteobacteria phylum is divided into six classes composed of Gram‐negative bacteria having the endotoxin lipopolysaccharide (LPS) in the outer membrane. Many common human pathogens are found in the Proteobacteria phylum, including the *Escherichia, Shigella, Salmonella, Yersinia, Helicobacter, Brucella*, and *Rickettsia* genera.^4^ Thus, when there is an overabundance of Proteobacteria at the expense of decreases in Firmicutes and Bacteroidetes, this is deemed a negative impact on the gut microbiome. The Actinobacteria phylum represents a small percentage of the gut microbiota and consists of Gram‐positive bacteria mainly represented by the *Bifidobacterium*, *Propionibacteria*, and *Corynebacteria* genera.^2^ Actinobacteria can produce SCFAs, and some *Bifidobacteria* species are used as probiotics to support gut health by promoting gut microbiome composition and function (Table 1).

**Table 1:** Human gut microbiota phyla and select genera/species.

Phylum	Description	Common genera/species
Firmicutes	One of the most abundant bacterial phyla in human gut is made up of Gram‐positive bacteria Comprising >200 different genera Some are beneficial with probiotic properties, and others are potentially pathogenic depending on the species	*Lactobacillus, Clostridium, Enterococcus, Ruminococcus, Faecalibacterium*, *Blautia*, and *Roseburia*
Bacteroidetes	One of the most abundant bacterial phyla in the human gut Made up of Gram‐negative bacteria Some are defined as beneficial, and some may be potentially pathogenic depending on the species	*Bacteroides, Prevotella, Porphyromonas, Alistipes, Parabacteroides, Barnesiella, Tannerella*, and *Capnocytophaga*
Proteobacteria	Found in small amounts in a healthy human gut Composed of Gram‐negative bacteria An unbalanced gut microbiome is often characterized by an overabundance of Proteobacteria	*Salmonella, Campylobacter, Helicobacter, Vibrio*, and *Escherichia*
Actinobacteria	One of the four major phyla in the human gut but only represents a small percentage Gram‐positive bacteria that help to maintain gut homeostasis	*Bifidobacteria, Propionibacteria, Corynebacteria, Streptomyces, Rothia*, and *Actinomyces*
Fusobacteria	Gram‐negative bacteria that can be beneficial or harmful depending on the species	Genera: *Fusobacterium, Leptotrichia, Ilyobacter, Propionigenium, Sebaldella, Streptobacillus*, and *Sneathia*.
		Pathogenic species: *F. necrophorum, F. nucleatum, F. canifelinum, F. gonidiaformans, F. mortiferum, F. naviforme, F. necrogenes, F. russi, F. ulcerans*, and *F. varium*
Verrucomicrobia	Gram‐negative bacteria considered essential for gut health	*Akkermansia muciniphilia, Prosthecobacter*, and *Verrucomicrobium spinosum*

**Figure 2:**
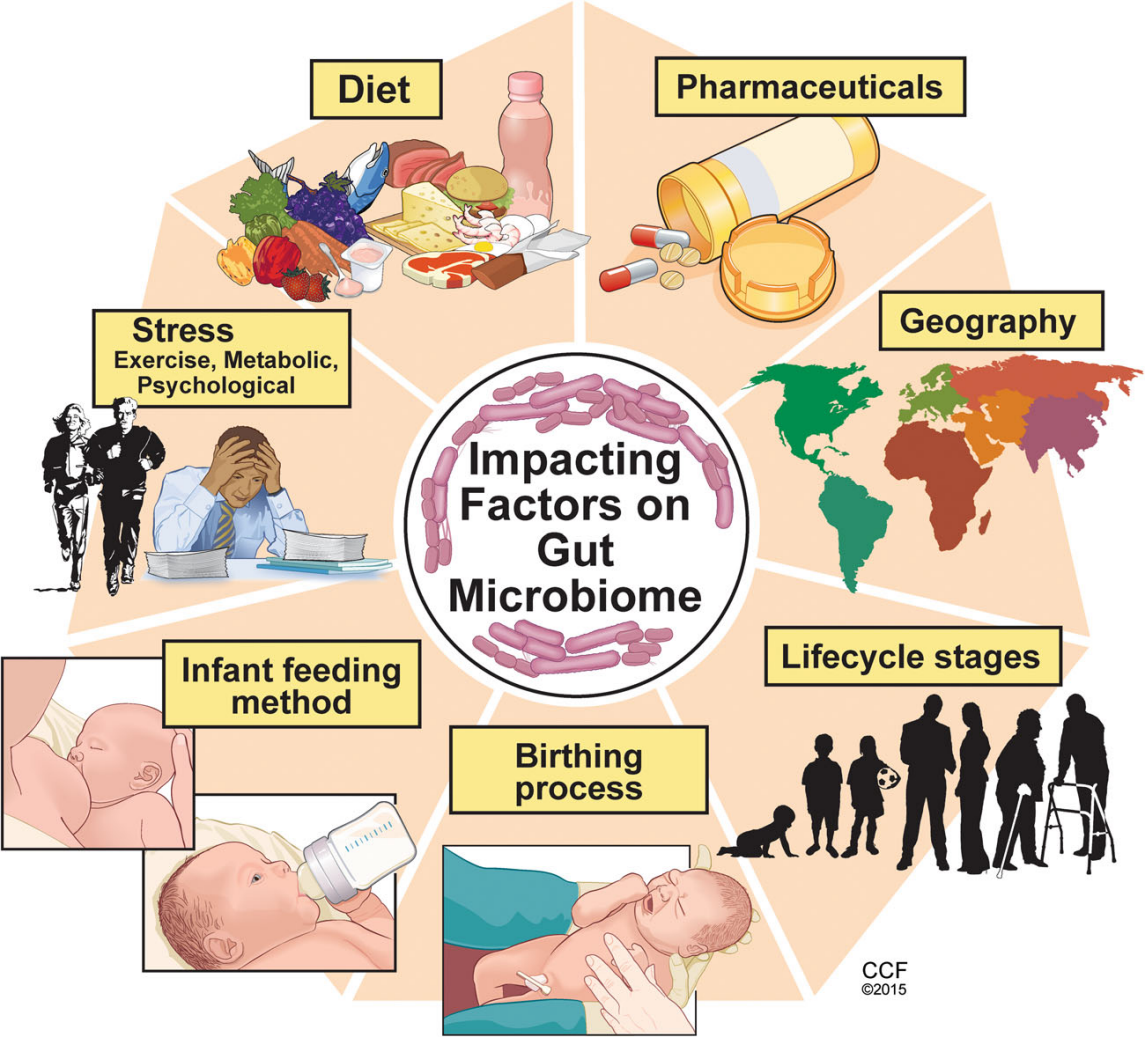
Factors impacting the gut microbiota. Many environmental and lifestyle factors can influence the composition and function of the gut microbiota.

The determination of what microbe composition defines a “healthy” gut microbiome is yet to be made. A more diverse and richer/complex (higher alpha diversity) microbiota is deemed more favorable as it has been related to improved resilience and resistance to change.^2^, ^4^ The gut microbiota varies between individuals (beta diversity), and variations may occur within the same individual. There is also variability in microbes within different locations of the gastrointestinal (GI) tract (Figure 1), largely because of differences in local environmental factors such as peristalsis, bile, and pH.^2^ Widespread studies have shown a key association between the gut microbiota and fundamental human biological processes, including energy and nutrient extraction from food, metabolism, biosynthesis of bioactive molecules, and developmental and protective immunity.^1^ Over the past several years, there has been a growing interest regarding the role of the gut microbiome, which is the gut microbiota including its genetic material, in supporting health. This interest has been sparked by the increasing number of chronic metabolic and inflammatory diseases that are associated with alterations in gut microbiome composition and function, often termed gut dysbiosis.^5^ Shaped from infancy, the gut microbiome rapidly changes in the first 2–3 years of life, after which it resembles that of an adult. These rapid changes are largely driven by the shift in infant diet from breastmilk or formula to a complex diet. The resulting ecosystem, influenced by sex, age, and ethnicity, is unique to the individual and has plasticity throughout life being impacted by environmental factors (Figure 2).^5^, ^6^

Most human data regarding the impact of diet on the gut microbiome are derived from healthy participants, and there is a gap in knowledge as to how different foods impact the microbiome of individuals with chronic diseases or those requiring enteral nutrition (EN) support. The complex nature of food makes it difficult to determine the causal nature of a particular dietary component on the gut microbiome. Hence, the purpose of this narrative review is to summarize the relevant research extracted from PubMed linking different macronutrients, food components, eating patterns, and enteral formulas and their impact on the gut microbiota. Relevant manuscripts in the English language have been selected using a combination of search terms in PubMed from 2009 to 2024 for each section. Keyword queries resulted in >10,000 manuscripts, and these were further selected based on their relevance to clinical nutrition and the gut microbiome as feasible. Key words included diet composition, high‐fat diet, low‐fat diet, vegan diet, plant‐based diet, omnivore diet, Western diet, carbohydrate, sugar, fructose, fiber, prebiotic, inulin, resistant starch, fruits, vegetables, phytochemicals, polyphenols, flavonoids, dietary fats, animal fat, plant oils, plant fats, polyunsaturated fats, unsaturated fats, monounsaturated fats, saturated fats, protein sources, meat, fish, chicken, nuts, peas, dried peas, soy protein, legumes, enteral formulas, enteral feedings, tube feedings, and EN.

All the above‐listed keywords have been used in combination with an “AND” builder with the following phrases: “gut microbiome” and “gut microbiota.” Both animal and human studies are included and in vitro studies in the cases where there was inadequate in vivo evidence. Despite that, we acknowledge that some studies may have been missed and may therefore not be included as this is not a systematic review.

## DIET AND THE MICROBIOME

### Energy distribution

Surpassing host genetics, diet is the key determinant of microbiota constitution, through modulation of the abundance of specific species and their combined functions.^7^, ^8^, ^9^, ^10^, ^11^ The gut microbiome is impacted by environmentally driven influences on dietary intake, such as seasonal variation and urbanization of the food supply,^8^, ^9^, ^10^, ^11^, ^12^, ^13^, ^14^ as well as the macronutrient composition of the diet. In healthy individuals, the gut microbiota shifts when there is a change in dietary macronutrients, and this occurs rapidly and reproducibly.^6^, ^9^, ^15^ These shifts are confounded by individual traits, such as sex, ethnicity, medications, and age, making it challenging to collectively evaluate changes.^5^, ^10^

Diet strongly affects human health, which may be due to gut microbiome composition and function. To determine the dietary impact on gut microbiome stability, a prospective randomized controlled‐feeding study with a high‐fat/low‐fiber or low‐fat/high‐fiber dietary intervention for 10 days was conducted in healthy participants.^10^ At baseline and study completion 10 participants provided stool samples, which were analyzed by 16S ribosomal RNA (rRNA) sequencing. Interparticipant variation was the most predominant source of variance in the data, and 10 days of controlled feeding of an identical diet did not overcome this. Individual participants’ microbiomes changed significantly within the first 24 h of starting the controlled feeding, and the taxa that changed differed among individuals indicating individualized microbial response to a change in diet. However, despite a detectable microbiome shift, enterotype identity, a classification of gut microbiota based on the relative abundance of certain microbes, remained stable during the 10‐day study supporting the notion that enterotypes are primarily influenced by long‐term diet and not a short‐term dietary change. Shotgun metagenomic analysis for total gene content showed that several bacterial functions responded to the high‐fat/low‐fiber or low‐fat/high‐fiber dietary interventions.

A recent study evaluated the effect of higher‐fat (40% of energy), moderate‐fat (30% of energy), and lower‐fat (20% of energy) diets on gut microbiota, fecal metabolomics, and circulatory inflammatory factors.^11^ In this 6‐month randomized controlled‐feeding trial in 217 healthy young Chinese adults (aged 18–35 years; body mass index [BMI] <28 kg/m^2^; 52% women), all foods were provided as isocaloric, isonitrogenous meals, the total fiber was kept at ~14 g/day, and the sources of macronutrients were similar between groups. Fecal samples collected at baseline and at 6 months showed a higher alpha diversity, increased abundance of *Blautia* and *Faecalibacterium* in the lower‐fat group, and increased *Alistipes* and *Bacteroides* and decreased *Faecalibacterium* in the higher‐fat group. Microbial functional changes also occurred between diet groups. Compared with the other groups, the predicted LPS biosynthesis and arachidonic acid metabolism pathways were increased, and fecal arachidonic acid was increased whereas SCFA levels were decreased in the higher‐fat group. Metabolites indole and p‐cresol, associated with metabolic disorders such as hypertension, cardiovascular disease, and chronic kidney disease, were decreased in the lower‐fat group. Together, these studies support that changes in dietary macronutrients from low to high fat can significantly shift the human gut microbiota composition and function. A high‐fat diet led to a less favorable microbiota with lower SCFA‐producing bacteria and associated metabolites and higher proinflammatory metabolites. This is unsurprising as gut microbiota use nutrients ingested by the host for their fundamental biological processes. Therefore, alterations in the host's diet can alter gut bacterial metabolism to favor bacterial species most suited to use the available fuel sources.

### Plant‐based vs animal‐based diets

Increased consumption of plants and plant‐based foods has been recommended because of this dietary pattern being associated with positive health outcomes and reduced disease risk.^12^, ^13^ In addition to health reasons, more people are consuming a plant‐based diet for environmental and ethical reasons. Most carbohydrates in plant‐based foods are metabolically available to the gut microbiota (microbiota‐accessible carbohydrate [MAC]). The microbiota of individuals who consume vegetarian or predominantly plant‐based diets exhibit greater capacity for MAC fermentation. Plant‐based foods also provide a vast array of phytochemicals that have the potential to affect human health. Owing to glycosylation, phytochemical bioavailability and bioactivity may be reduced, allowing MAC to reach the distal gut. Here phytochemicals can be modified by gut microbial enzymes into metabolites with increased bioavailability and altered bioactivity.^14^ Polyphenols, the most diverse group of phytochemicals, have been shown to undergo transformation into beneficial metabolites by gut microbes, and likewise, polyphenols have been shown to favorably transform the microbiome.^15^ An example of polyphenol‐microbiome interactions is with resveratrol and curcumin, where these polyphenols with anti‐inflammatory properties were shown to impact *Bifidobacterium* and gut microbial pathways controlling carbohydrate, sulfur, and amino acid metabolism while improving glycemic control in mice.^16^ These data suggest that resveratrol's known beneficial effects on glycemic control as an adjunct treatment for patients taking metformin^17^ may be through its modulation of the gut microbiome's metabolic activity.

Long‐term consumption of a plant‐based diet has also been shown to influence the gut microbiome, increasing taxonomic and bacterial gene diversity, SCFA, and the *Prevotella/Bacteroides* ratio.^18^, ^19^ A study analyzed the microbiome and metabolome in those who consumed a plant‐based/vegan (*n* = 15) or omnivore (*n* = 16) dietary pattern for a minimum of 6 months and resided in an urban environment in the Northeastern US.^18^ Despite clear differences in dietary patterns, including more carbohydrate and less protein and fat in the plant‐based diet consumers, there were no discernable differences in taxa at the genus level between the diet groups via 16S rRNA sequencing. However, of the 361 plasma metabolites tested, 96 (25%) differed between the omnivores and vegans. Lipid and amino acid metabolites were elevated in the omnivores, and xenobiotic (a chemical substance foreign to a living organism) metabolites were elevated in the vegans. A multivariate analysis was used to identify whether biochemicals separated in participants according to their diet. Thirty metabolites grouped into six areas (amino acids, carbohydrate, cofactors and vitamins, lipid, nucleotides, and xenobiotics) were identified with a 94% predictive accuracy. Urine metabolomics also revealed metabolite separation between the dietary patterns. But, unlike the diet, the gut microbiota composition was not associated with plasma metabolome. However, relative to the omnivore diet, the vegan diet resulted in higher plasma metabolites derived from plants, such as ascorbate, xanthine metabolites, and products of benzoate metabolism. Foods common in a vegan diet that are rich in phenolic phytochemicals (eg, berries, nuts, and grains) could be the source of these metabolites that were modified by the gut microbiome as was previously described.^15^

David et al evaluated the short‐term gut microbiota response to a rapid change in plant‐based vs animal‐based diets.^9^ Each diet was consumed by 10 healthy volunteers (6 males aged 21–33 years) ad libitum for 5 consecutive days, which was preceded by 4 days of the volunteer's typical diet consumption run‐in (baseline) and a 6‐day recovery (washout). Each diet arm significantly shifted volunteers' macronutrient intake with increases in dietary fat by 37%, dietary protein by 15%, and a decrease in fiber by 9 g/1000 kcal to almost no fiber intake on the animal‐based diet. Both fat and protein intake decreased to 22% and 10% of energy, respectively, and fiber rose to 25 g/1000 kcal on the plant‐based diet. Gut microbiome composition assessed using 16S rRNA sequencing found no differences in alpha diversity (intraindividual) when volunteers were on either diet; however, there was a significant increase in beta diversity (interindividual) that was unique to the animal‐based diet. This change occurred within a single day of food reaching the distal gut as evidenced by a food‐tracking dye. The gut microbiota reverted to its original structure within 2 days of finishing the animal‐based diet. The animal‐based diet altered the relative abundance of bacterial taxonomic groups more so than the plant‐based diet. Interestingly, volunteers’ fiber intake over the prior year correlated positively with baseline *Prevotella* levels, a taxon posited to be sensitive to long‐term fiber intake. The animal‐based diet resulted in lower products of carbohydrate fermentation and higher products of amino acid fermentation, which correlated with saccharolytic and putrefactive microbes, respectively, suggesting that the macronutrient shifts also altered gut microbiota function.

## DIETARY FOOD SOURCES AND THE GUT MICROBIOME

### Carbohydrate

Many dietary carbohydrates, or glycans, are resistant to host digestion and present in many forms, including long polysaccharide chains (eg, cellulose, pectin, and resistant starch), oligosaccharide chains (resistant to host digestion) that are linked to proteins or lipids (eg, glycoproteins or glycolipids), and sialic acids.^20^ Host‐digestible monosaccharides and disaccharides, either alone or in combination with a Western‐style high‐fat diet, have been shown to negatively influence the gut microbiome by specifically inhibiting colonization with beneficial symbiont bacteria.^21^, ^22^ Colonization of *Bacteroides thetaiotamicron*, a well‐characterized bacteria shown to reduce colonization of pathogenic microbes in gnotobiotic mice, was inhibited by dietary glucose and fructose's downregulation of a protein, regulator of colonization.^23^ High fructose intake induces hepatic steatosis in both human studies and mouse models, and this occurrence is correlated with reduced gut microbiota abundance of *Bifidobacteria*, *Lactobacillus*, *Bacteroides*, and *Ruminiococcus*.^22^

Complex carbohydrates with their diverse array of monosaccharide linkages may not fully be accessible to host enzymes and therefore escape digestion and become accessible to the gut microbiota. Evidence suggests that depletion of complex carbohydrates from the diet affects gut microbiota composition and function.^24^, ^25^ Mice colonized with human microbiota and consuming a low complex carbohydrate diet had a loss in microbe diversity, which was compounded over several generations of offspring, which did not recover after reintroduction of complex carbohydrates into the diet.^25^ To restore the microbiota to its original composition, missing taxa were required to be provided along with the complex carbohydrates.^25^

### Dietary fiber and prebiotics

Most of the information relating to carbohydrate and the gut microbiome pertains to dietary fiber (Table 2). Dietary fiber is generally thought of as edible polysaccharides, sourced primarily from plants, that are not digestible by host enzymes. There are soluble and insoluble forms of dietary fiber, although some sources can be both, depending on cooking or food processing.^26^ As humans are unable to digest fiber, it reaches the distal gut and interacts with certain species within the gut microbiota that possess enzymes capable of digesting or fermenting these polymers.

**Table 2:** Types of dietary fibers.^26^

Chain length	Solubility	Fermentability	Examples
Short chain	Soluble oligosaccharides,	Highly fermentable	Fructooligosaccharide and galactooligosaccharide (raffinose and stachyose)
Long chain	Soluble nonstarch polysaccharides	Highly fermentable	Resistant starch, pectin, inulin, and guar gum
	Intermediate soluble and fermentable fiber	Intermediate fermentable	Psyllium/ispaghula and oats
	Insoluble	Slowly fermentable	Wheat bran, lignin (flax), fruits, and vegetables
	Insoluble	Nonfermentable	Cellulose, sterculia, and methylcellulose

Short‐chain fibers include the oligosaccharides, which are highly fermentable compared with the longer‐chain fibers. The longer‐chain fibers can be classified into four main groups based on their solubility and fermentability, as listed in Table 1. Upon fermentation by the gut microbiota, fermentable fibers yield energy and metabolic substrates. Additionally, the presence of these fibers in the distal gut may positively support or influence changes in the gut microbiota composition and function. As such, some fibers have been classified as dietary prebiotics, “a selectively fermented ingredient that results in specific changes in the composition and/or activity of the gastrointestinal microbiota, thus conferring benefit(s) upon host health.”^27^

Prebiotics naturally exist in a variety of foods (Table 3). However, as the concentrations of prebiotics in these sources may not be enough to exert a prebiotic effect, some are manufactured on industrial large scales. The commercial market has been dominated by a few prebiotics, mainly inulin, fructooligosaccharides (FOSs), and galactooligosaccharides (GOSs), as well as a few isomalto‐oligosaccharides.^28^ These prebiotics may be added to a wide variety of processed foods and beverages. Infant formulas are often supplemented with GOSs, FOSs, and selected human milk oligosaccharides as a means to induce *Bifidobacteria* in a similar manner as human milk oligosaccharides in breastfed infants.^29^ Inulin and FOS may also be added to enteral tube feeding formulas to minimize diarrhea and support gut microbiome composition and function.^30^ The degree of polymerization (DP) of the prebiotic helps to distinguish which microorganisms may be capable of fermenting it. For example, inulin with a DP of ≤60 can only be fermented by a few species, whereas FOS with a DP of ≤10 can be fermented by a multitude of microbes.^31^

**Table 3:** Food sources of prebiotics.

Food group	Foods
Vegetables	Asparagus
	Sugar beet
	Garlic
	Chicory
	Onion
	Jerusalem artichoke
	Tomato
	Peas
	Corn
	Jicama
	Eggplant
	Raw leafy greens (eg, dandelion, leak, and endive)
Grains	Wheat
	Barley
	Rye
	Oats
Fruits	Green banana
	Apples
	Berries
Nuts and legumes	Soybean
	Dried beans
	Almonds
	Flaxseed
Sugars	Honey
	Agave
Dairy	Human milk
	Cow's milk

As fiber is the preferred food for the gut microbiota, when it is lacking in the diet the gut microbes must forage for their energy supply. The colon has two layers of mucus, a loose luminal outer layer and a dense inner layer.^32^ In a healthy person, the gut microbes do not penetrate the inner mucus layer, but the outer layer is degraded by gut microbes as part of normal mucin turnover and regeneration. However, excessive mucin degradation is associated with bacterial penetration into the inner mucus layer and colonic inflammation.^33^, ^34^, ^35^, ^36^, ^37^, ^38^, ^39^ Mucin is composed of host mucin proteins and regions of extensive *O*‐glycosylation. Studies show that bacteria shift to metabolize colonic mucin when dietary fiber is lacking. A study using gnotobiotic mice showed that in the absence of a dietary supply of polysaccharides, *B. thetaiotamicron* redirects itself to host glycans to find a suitable food supply.^25^ Other microbes, such as *Akkermansia mucinophila*, can degrade host mucins but not dietary fiber, and thus expand their population when dietary fibers are scarce.^34^ Mice fed a low‐fiber Western diet demonstrated both altered gut microbiota composition and decreased growth of the inner colonic mucus layer. This was reversed when mice received a fecal transplant from chow‐fed mice. Inulin supplementation in mice prevented mucus penetration with bacteria.^35^ Desai et al showed that dietary fiber deprivation in gnotobiotic mice colonized with human microbiota caused the gut microbiota to use host‐secreted mucus glycoproteins for its nutrition, which eroded the colonic mucus barrier and enhanced pathogenic bacteria colonization.^20^ Taken together, these studies highlight the importance of dietary fiber in supporting the gut microbiome and intestinal integrity. The extent of microbial mucus foraging in humans and its importance to human disease has not been fully explored.

### SCFAs

SCFAs, such as acetate, propionate, and butyrate, are beneficial metabolites of dietary fiber fermentation by the gut microbiota. SCFAs are absorbed by the intestinal epithelial cells, where most of the butyrate is used directly as an energy source for the colonocyte, or they enter circulation where they are further metabolized in the liver, muscle, or other peripheral tissues and thought to contribute 7%–8% of host daily energy requirements.^31^ Of the SCFAs, acetate is in the highest concentrations in the gut lumen and blood followed by propionate, which contributes to gluconeogenesis in the liver and promotes satiety and reduces cholesterol.^36^, ^37^ Butyrate is known to have anti‐inflammatory and immunomodulatory effects and to induce epigenetic changes to DNA via its histone deacetylase inhibitor activity.^38^ The different types of fibers that reach the distal gut for fermentation are dependent on their daily ingestion, and not all dietary fibers yield SCFA equally.^37^ A diet high in whole fruits, vegetables, and grains would yield higher levels of SCFAs than one low in these foods. Because it is difficult to study the direct effects of SCFA's biological roles together or individually in humans, most available data comes from preclinical experimental models.^39^

There are differential responses to SCFA production based on individual factors. Individuals with obesity and genetically obese mice show increased fecal and luminal levels of SCFA. This has led to the suggestion that the SCFAs may contribute to an increased ability to absorb energy from the diet.^40^ A person's baseline microbial richness has been shown to influence its resilience to change with a dietary fiber adjustment, with a higher richness less responsive to change. Looking at one genera, changes in *Bifidobacteria* abundance were shown to be influenced by an individual's baseline levels, with lower *Bifidobacteria* levels being more responsive to change with a dietary adjustment.^31^, ^41^ Also, an individual's habitual diet can impact the way their microbiome may respond to dietary fiber manipulation. Healey et al conducted a randomized, double‐blind, placebo‐controlled, crossover study in 34 healthy participants.^42^ Participants were classified as consuming low‐fiber (<18 g/day for women and <22 g/day for men) vs high‐fiber (≥25 g/day for women and ≥30 g/day for men) groups based on their habitual dietary intake of fiber. Participants received either 16 g/day powdered inulin‐type fructan or 16 g/day powdered placebo (maltodextrin) as two 8‐g/day doses for 3 weeks. There was a 3‐week washout period between supplements. In the low‐fiber group, the only significant change in microbiome was an increase in the relative abundance of *Bifidobacterium*, whereas the high‐fiber group had increased *Bifidobacteria and Faecalibacteria* relative abundance and decreased relative abundance of *Coprococcus*, *Dorea*, and *Ruminococcus*. These data suggest that those with a habitual high‐fiber intake were more likely to have a microbiota response to prebiotic supplementation than those with low‐fiber intakes.

## IMPACT OF CARBOHYDRATE‐CONTAINING FOODS ON GUT MICROBIOTA

### Whole grains

In addition to a mixture of cellulose, resistant starches, and oligosaccharides, whole grains contain unique hemicellulose fibers, such as xylans and β‐(1 → 3, 1 → 4) glucans. In animal studies, whole cereal grains compared with refined grains increased the diversity of the gut microbiota and taxa abundances of *Prevotella* and *Anaeroibrio*.^43^ Human studies also show beneficial effects of whole‐grain ingestion on microbiome composition and function. A study in which the recommended daily intake of β‐glucan (3 g/day) was consumed for 2 months by healthy individuals via durum wheat flour and whole‐grain barley pasta showed an induction of *Clostidiaceae*, *Roseburia hominis*, and *Ruminococcus* spp as well as SCFA levels.^44^ Consumption of whole barley, brown rice, or a mixture for 4 weeks in healthy people increased gut microbiota diversity, the Firmicutes:Bacteroidetes ratio, and the abundance of *Blautia* and *Eubacterium rectale* genera.^45^ Interestingly, gut microbiota enzymes key in carbohydrate digestion, glycoside hydrolases, increased with 3 days of increased dietary intake of whole barley and was associated with improved glucose tolerance in the responders.^46^ However, several studies show only a modest or no change in gut microbiota with whole‐grain consumption.^47^, ^48^ Overall, these studies suggest that the impact of dietary intake of whole grains may be dependent on several factors, including habitual intake of fiber, other dietary factors, and baseline microbiota.

### Fruits and vegetables

Fruits and vegetables provide up to 8 g of dietary fiber per serving^49^ and contain a mixture of insoluble, soluble, and fermentable fibers.^50^, ^51^ Different sources of dietary fibers exert distinct effects on the gut microbiota. For instance, pectin is a soluble fiber contained in many fruits and vegetables. Apple pectin was shown to increase *Clostridiales* and decrease *Bacteroides* spp abundance in rats.^52^ Citrus pectin increased the abundance of Bacteroidetes,^53^, ^54^ and *Faecalibacterium prausnitzii* was increased with apple‐, but not citrus‐derived, pectin.^55^ In rats, fecal abundance of *Bacteroides*, *Prevotella*, and *Porphyromonas* was increased with broccoli fiber, inulin, potato fiber, and potato‐resistant starch, whereas gut pathogenic bacteria (*Clostridium perfringens*, *Escherichia coli [E coli]*, and *Enterococcus* spp) were decreased with broccoli fiber and inulin.^50^ Thus, these data suggest that a variety of fibers provided in fruits and vegetables can help to maintain a diverse microbiome.

### Phytochemicals

In addition to fiber, fruits and vegetables are also rich sources of phytochemicals, including polyphenols, glucosinolates, terpenoids, phytosterols, and alkaloids, which have been shown to modify the gut microbiome.^56^ Cranberry extract, a rich source of polyphenols, was shown to increase the commensal microbe *Akkermansia muciniphila*, which was related to a reduction in weight gain, visceral adiposity, hepatic steatosis, oxidative stress, and inflammation induced by a high‐fat, high‐sucrose diet fed to mice.^57^ In humans, *Bifidobacteria* abundance was enhanced with the consumption of a wild blueberry powdered drink for 6 weeks,^58^ and consumption of red wine was associated with increased *Bifidobacteria, Bacteroides*, and *Prevotella*.^59^ SCFAs were increased, and endotoxin was decreased in a study in humans providing polyphenol‐rich mango.^60^

Flavonoids, present in fruits, vegetables, legumes, nuts, and seeds, have been shown to have positive effects on the gut microbiota by increasing the production of SCFAs and reducing systemic endotoxin. Anthocyanins extracted from blueberries or grapes significantly enhanced the beneficial taxa *Lactobacillus*, *Enterococcus* spp, and *Bifidobacterium* spp.^61^, ^62^ When consumed for 3 weeks, pomegranate extract, which contains both polyphenols and flavonoids, induced the abundance of *Faecalibacterium, Odoribacter*, and *Parvimonas* and reduced endotoxin.^63^ Together, these data suggest that the consumption of a variety of phytochemicals, contained in fruits and vegetables, can have a beneficial effect on the gut microbiome.

## FAT SOURCES ON GUT MICROBIOTA

The type and quantity of dietary fat impacts bile composition and secretion, and fractions of unabsorbed fat can reach the colon and influence the composition and metabolic activities of the gut microbiota. As previously discussed, high‐fat diets are frequently shown to increase the abundance of the Firmicutes phylum compared with low‐fat diets. However, not as many studies have compared the specific sources of dietary fat on the gut microbiota. Fat sources also vary in other components they contain, which can have an impact on the gut microbiota. For example, avocados are rich in fiber, whereas vegetable oils contain phytochemicals, including polyphenols.

A randomized control trial conducted for 12 weeks in overweight adults compared the consumption of an isocaloric meal with one that contained fresh Hass avocados, 175 g/day for men or 140 g/day for women, found an increased fecal alpha diversity the relative abundance of genera *Lachnospira*, *Alistipes*, and *Faecalibacterium*; and acetate, but diminished relative abundances of *Roseburia* and *Ruminococcus*.^64^ Another study testing for microbiome and inflammatory markers in participants who were healthy and obese/overweight were randomized to consume an avocado hypocaloric diet vs a hypocaloric diet without avocados for 12 weeks. Those who consumed the avocados had decreased blood inflammatory cytokine markers and C‐reactive protein, which was associated with significant changes in the relative abundance of *Bacteroides*, *Clostridium, Methanospaera*, and *Candidatus Soleaferrea* genera.^65^

Olive oil has varying degrees of polyphenol content based on its processing method, with virgin olive oil having the highest content. A study in mice fed a standard diet (3% energy as fat) or high‐fat isocaloric diets (35% energy as fat) enriched in extra virgin olive oil (EVOO) or butter tested whether several bacterial taxa were correlated with markers of metabolic syndrome. Mice receiving butter had the highest systolic blood pressure, which positively correlated with *Desulfovibrio*. The EVOO group had the lowest plasma insulin, which showed an inverse relationship with *Desulfovibrio*.^66^ Another study compared mice fed a high‐fat (40% of energy) diet composed of different sources of fats (olive oil, corn oil, or milk fat) vs a low‐fat chow (9% energy with corn oil) diet group for 5 weeks and found the high‐fat diets displayed increased abundances of the Firmicutes phylum.^67^ Increased abundances differed based on the source of fat. Olive oil increased *Clostridiaceae, Peptostreptococcaceae, Ruminococcaceae*, and *Dorea* spp. Milk fat increased the *Erysipelotrichales* and *Ruminicoccus* genera, and corn oil increased the *Turicibacteracea* and *Coprococcus* spp. The milk fat group had similar SCFA levels to the low‐fat chow group compared with both oil groups, for whom SCFA levels were reduced.

Plant sources of saturated fats, palm and coconut oils, have been studied in animal models for their effects on gut microbiota. Higher‐quality (virgin) coconut oil compared with standard chow, which contains soybean oil as the primary fat source, provided for 16 weeks increased the abundance of *Lactobacillus*, *Allobaculum*, and *Bifidobacterium* species and improved type 2 diabetes mellitus parameters in rats.^68^ In a human study of healthy volunteers, increased oxidative stress but lowered blood cholesterol resulted in those consuming a 15% polyunsaturated fatty acid (PUFA) vs a 5% PUFA‐containing diet for 4 weeks.^69^ Negative effects of fish oil feeding were found in rodent studies. Different gut microbiota structures occurred in middle‐aged rats fed lard, fish oil, or soybean oil (4% wt/wt) for 3 months.^70^ The composition of the gut microbiota in the fish oil group varied from the soybean and lard‐fed groups. The fish oil–fed group exhibited a higher abundance of Proteobacteria phylum and genus *Desulfiovibrio*, which was associated with increased gene expression of inflammatory markers in the colon. Thus, these data suggest that the negative effects of fish oil on inflammation and oxidative stress observed could be a consequence of dietary fat sources on gut microbiota alterations.

In a double‐blinded randomized crossover study, the effects of five different oil blends fed as part of a 7‐day rotation isocaloric menu for 30 days each were tested in healthy volunteers at risk for metabolic syndrome.^71^ Fat‐blend treatments consisting of 60 g/day included three monounsaturated fatty acid (MUFA)–rich diets (conventional canola oil, docosahexaenoic acid–enriched high‐oleic canola oil, and high‐oleic canola oil), and two PUFA‐rich diets (corn/safflower oil blend [25:75] and flax/safflower oil blend [60:40]). Stool samples were collected and analyzed at the end of each period. The study results showed that the oil blends did not alter the bacterial phyla; however, a higher Firmicutes:Bacteroidetes ratio occurred in those with obesity compared with those who were overweight or normal weight. Similarly, genus‐level microbiota changes were related to BMI classification. There were differences between the PUFA‐ and MUFA‐rich diets. The MUFA‐rich diets increased *Parabacteroides, Prevotella*, *Turicibacter*, and *Enterobacteriaceae* abundances, and the PUFA‐rich diets increased the abundance of *Isobaculum*. In people with obesity, the MUFA‐rich diets increased *Parabacteroides* and decreased *Isobaculum*. These data suggest that microbiota profiles differ among BMI classifications and that dietary fat composition impacts gut microbiota composition at lower taxonomical levels in those with obesity.

## PROTEIN AND THE GUT MICROBIOTA

Although the protein contained in foods is digested and absorbed in the proximal intestine by the host, gut microbiota in the small intestine can also metabolize dietary protein. Dietary protein is a primary source of amino acids for intestinal microbiota, where it can be used for protein synthesis and metabolic energy. The small intestinal bacteria reported to metabolize proteins or secrete proteases and peptidases include *Klebsiella* spp, *E coli*, *Streptococcus* spp, *Succinivibrio dextrinosolvens*, *Mitsuokella* spp, *Anaerovibrio lipolytica*, and *Lactobacillus johnsonii*.^72^ For example, *L. johnsonii*, a commensal microbe within the small intestine, lacks the gene encoding for biosynthetic pathways for amino acid production. However, *L. johnsonii* produces an extracellular protease, oligopeptide transporters, ≥25 cytoplasmic peptidases, and 20 amino acid‐permase type transporters, suggesting its dependence on the host or other intestinal microbes to provide its nutrients. This information suggests that not all dietary protein is available to the host. This may be of particular interest when there is an overabundance of protein‐preferring microbes in the small intestine, such as in critical illness and when high loads of dietary protein are provided. Whether dietary protein is being used by gut microbes for their metabolic support and survival during critical illness warrants further study.

An ample quantity of undigested amino acids may enter the colon and interact with the gut microbiota to be fermented into various intermediary or end‐product metabolites such as SCFAs, hydrogen sulfate, polyamines, ammonia, and phenolic and indolic compounds.^73^, ^74^ These bacterial metabolites can be transported into colonocytes and exert beneficial or deleterious effects on epithelial cells depending on their luminal concentration. Some amino acid metabolites are transported to the liver or peripheral tissues to have various physiological effects. Several genera present in the colonic microbiota have been shown to possess proteolytic activity, including *Bacteroides, Propionibacterium, Streptococcus, Fusobacterium, Clostridium*, and *Lactobacillus*.^74^ *Bacteroides* species are present in the small intestine and colon and can secrete proteases. An overabundance of *Bacteroides* can result in an excess of proteases, which may be able to degrade maltase and sucrase enzymes in the brush border of enterocytes.^75^ High amounts of dietary protein can influence the gut microbiota as the unabsorbed residual nitrogenous compounds in the small intestine will move to the colon to be metabolized by the microbiota. Excessive protein supplementation can result in an increased abundance of potentially pathogenic microbes because of disruption in homeostasis of the gut ecosystem.^73^ Evidence derived from studies with mono‐gastric livestock leads to recommendations for lower concentrations of dietary protein for animal health to reduce the amount of substrate for pathogenic bacteria proliferation. However, too low dietary protein can also increase the abundance of pathogenic bacteria, so finding the right balance is essential.^76^

### Dietary protein sources and the gut microbiota

Zhu et al tested the effects of different protein sources on gut microbiota in rats.^77^ Isocaloric, isonitrogenous diets differing only in the source of protein (beef, chicken, pork, fish, casein, or soy) were fed to rats for 90 days, and then euthanasia cecal samples were analyzed via 16S rRNA for microbiome profiles. In response to dietary proteins, there was substantial intragroup and intergroup variation of gut bacteria. By Bray‐Curtis analysis, samples were clustered into two groups: nonmeat (casein and soy proteins) and meat (fish, chicken, beef, and pork). Although Bacteroidetes and Firmicutes were the predominant phyla in all six protein groups, at the phylum level the six groups formed three clusters: (1) casein and soy, (2) pork and beef, and (3) chicken and fish. The commensal *Lactobacillus* genera was higher in white meat than in the red meat or nonmeat protein groups. Blood LPS‐binding protein, a marker for antigen load, was lower in rats fed meat proteins and casein, suggesting these protein sources maintained a more balanced gut microbiota balance that facilitated lower antigen load and inflammatory potential.

Dietary protein sources vary in the types and amounts of fat they contain, which may also impact the gut microbiota. Lang et al randomized healthy adults (*n* = 109) to either high (15% of total energy) or low (7% of total energy) saturated fat groups. These participants randomly received three diets composed of different protein sources for 4 weeks each.^78^ Protein provided 25% of the total energy, of which all groups received 10%–13% of energy with dairy foods and eggs. The remaining protein was provided as red meat (12% of energy; beef and pork), white meat (12% of energy; chicken and turkey), and nonmeat (15% of energy; nuts, beans, and soy). For a washout period between diet groups, the participants consumed their usual diet for 2 weeks. The 16S rRNA sequencing and analysis of gut microbiota found the different diets caused modest changes in the gut microbiota. Saturated fat intake level was more influential than the protein source on taxon abundance. Accounting for sex, age, ethnicity, and diet order, there were 151 differentially abundant operational taxonomic units (OTUs; a group of bacteria that are closely related and grouped together based on the similarity of their DNA structure) between the high and low saturated fat groups, and three OTUs were differentially abundant between the various protein diets. However, when assessing the effect of the source of protein once the data were analyzed separately for high and low saturated fat levels, it was found that the protein source influenced the microbiome, with more OTUs differentially abundant in the high saturated fat group. Of the common OTUs, 19 were consistent between the low and high saturated fat levels. These taxa, which included *Bacteroides* and *Sutterella*, were termed “protein‐sensitive” OTUs because they responded regardless of saturated fat level. Interestingly, the most influential factors on the microbiome were traits describing interindividual variation. For example, sex differences accounted for 84 differentially abundant OTUs when age and ethnicity were adjusted. Men had a significantly higher Bacteroidetes:Firmicutes ratio on the baseline diet and the experimental diets. Ethnicity also impacted alpha diversity (Shannon index) when both the baseline and experimental diets were consumed by White, Asian, and African American participants. Thus, these results suggest that moderate changes in the percentage of dietary saturated fat and protein sources led to modest changes in the microbiome in healthy participants and that interindividual traits provide important input into how these diet factors impact the gut microbiota.

Increased consumption of plant‐based protein has gained consumer interest for both health and environmental reasons. Compared with animal protein, plant protein typically has lower digestibility associated with the undigestible cell wall of the plant (fiber). Likely because of the lack of plant cell wall fiber, ingestion of animal protein is characterized by a reduction of SCFAs and an increase in gut pH and ammonia concentration.^79^ *Glycine max*, commonly called soybean, is an important plant‐based protein source containing all nine essential amino acids in quantities that can meet human physiological requirements.^80^ The amount of protein in soybeans is almost double that found in commonly consumed beans and legumes, and the leucine content is comparable to amounts found in fish and eggs. Like other consumed beans, soybeans are a good source of fiber (oligosaccharides and nonstarch polysaccharides), PUFAs, and micronutrients (calcium, iron, and zinc), and they contain isoflavones.^80^ Data for the effect of soy protein on the gut microbiome come from experimental models. Studies in rodents have shown that dietary interventions with soy protein compared with casein increased bacterial diversity and altered specific bacterial species, but changes vary between studies.^80^ Increasing the soy protein isolate content from 15% to 25% increased SCFA levels in rats compared with those fed the same amount of casein protein.^81^

Pea protein is made up of 15%–25% pea albumin and 50%–60% pea globulin, with a high lysine and tryptophan content.^82^ Peas (*Pisum sativum*) are also rich in fiber (hull fiber, resistant starch, and oligosaccharides), carbohydrate, ferritin, vitamins, minerals, and phytochemicals.^82^ Although the bioactive peptides of peas contribute to their health benefits, more recently the gut microbiome modulation by pea protein has also become of interest. An in vitro study with simulated GI digestion and healthy human gut microbiota tested the effect of hydrolyzed pea protein isolate on microbiota composition and function.^83^ Pea protein isolates and hydrolysates from golden field peas increased total aerobic and anaerobic bacteria load and SCFA production relative to a pea protein–free control. In the in vitro system, pea protein isolates led to higher levels of *Bacillaceae, Bacteroidaceae, Porphyromanodaceae, Lachnospiraceae*, and *Coribacteriaceae*. Because of their high lysine content, pea proteins are susceptible to spontaneous glycation during storage and cooking. As glycation can alter the structure of food macromolecules making them highly bioactive, another study tested how glycation of pea protein affected bacterial adhesion on intestinal enterocytes. Using in vitro models of the human GI tract and gut microbiota by incorporating human feces and digestive enzymes, both pea protein and glycated pea protein enhanced the adhesion of beneficial bacteria to intestinal enterocytes.^84^ Glycated pea proteins increased the proliferation of intestinal bacteria compared with nonglycated pea protein and control culture. Both glycated and nonglycated pea protein stimulated the growth of genera *Bacteroides*, *Lactobacillus/Enterococcus, Clostridium* (*C. perfringens/histolyticum* subgroup), and *Bifidobacterium* up to the eighth hour of culture as determined by fluorescence in situ hybridization (FISH) analysis.^85^ A crossover study reported diverse responses in the gut microbiome in hamsters fed pea protein vs pork protein that was related to a cholesterol‐lowering effect with pea protein.^86^ The pea protein group had a low Firmicutes:Bacteroidetes ratio and enhanced abundance of *Muribaculaceae* and *Ruminococcaceae*, whereas the pork protein group had increased *Erysipelotrichaceae* and *Eubacteriaceae*. Pea protein also altered cecal metabolites, including metabolites within the arginine/histidine pathway, primary bile acid biosynthesis, SCFA, and other lipid‐like molecules involved in cholesterol metabolism. Furthermore, when animals were treated with antibiotics, the differences in serum or liver cholesterol were eliminated, suggesting gut microbiota involvement in cholesterol metabolism. In summary, the effects of protein sources on the gut microbiota are not consistent, and more studies are needed to evaluate the effects of different protein types on protein digestibility, metabolism, and gut microbiota composition and function.

## ENTERAL TUBE FEEDING FORMULATIONS AND THE GUT MICROBIOME

EN provides nutrition either orally or through a feeding tube for people unable to consume adequate food or nutrients in their diet.^87^ Patients may be prescribed EN to fully or partially meet their nutrient requirements. In the US, it is estimated that >250,000 malnourished hospitalized patients receive EN,^87^, ^88^ and in Europe and Japan, approximately 10% of hospitalized patients receive EN.^89^ Although many patients receiving EN have suffered an acute injury, they may also have chronic diseases such as inflammatory bowel disease, cardiovascular disease, diabetes mellitus, and metabolic syndrome. All these conditions are associated with gut dysbiosis.^90^, ^91^, ^92^, ^93^ Critical illness results in gut dysbiosis, as early as 6 h of the inciting event.^94^ Therapeutic interventions during critical illness, such as antibiotics, contribute to the rapid reduction of commensal and overabundance of potentially pathogenic microbes. This gut microbial imbalance may be further exacerbated by starvation; oxidative stress; delivery of medications, such as gastric acid suppression agents, steroids, and antipsychotics; and parenteral nutrition or EN that lacks soluble fibers.^89^, ^94^

Although the provision of EN is the preferred feeding route, EN may also be associated with GI complications such as bloating, gas, vomiting, constipation, and diarrhea. In the critically ill, studies have examined the impact of EN composition on the development of diarrhea. Various aspects of EN have been associated with developing diarrhea including high osmolality, high fiber content, and high protein‐containing formulas.^95^ A meta‐analysis of 13 studies of critically ill patients (*n* = 709 patients) assessed the effects of dietary fiber on enteral feeding intolerance and clinical outcomes.^96^ Nine of the 13 studies (*n* = 553 patients) measured diarrhea as an outcome. The group receiving fiber had a significantly reduced risk of diarrhea compared with that of the fiber‐free group (odds ratio = 0.46; 95% CI, 0.30–0.69; *P* < 0.001; *I* ^2^ = 33%). Additionally, compared with the fiber‐free group, the group receiving fiber had a significantly reduced risk of regurgitation, vomiting, and constipation. Fiber provision was also associated with a reduction in ICU and hospital length of stay, and fiber provision did not impact the risk of mortality. Ni et al evaluated differences in the gut microbiota in critically ill patients receiving EN with or without diarrhea.^89^ Patients enrolled were similar in the antibiotics they received, did not have hypoalbuminemia, did not receive potassium, gastric acid reducing, or prokinetic medications, did not recieve probiotic supplements, and patients were not severely malnourished. Information regarding the EN formulation provided and whether it contained fiber was not provided. Compared with those without diarrhea, EN patients with diarrhea had differences in bacterial composition and significantly decreased bacterial richness and diversity. Moreover, KEGG (biochemical) pathways related to immunity and metabolism were altered in EN patients with diarrhea. Together these data suggest that EN containing fiber is not harmful for critically ill patients and may improve common GI symptoms. Whether improvement in GI symptoms is through EN with soluble fiber favorably impacting the disrupted gut microbiota in critical illness warrants further study.

Standard enteral feeding solutions are highly processed, commercially sterile food‐like substances formulated to provide full nutrient requirements when the recommended volume is provided. Macronutrient sources may vary depending on whether the formula is intended for oral consumption or administration via a feeding tube, and some formulations include added fibers (see Table 4). Additionally, there are food‐based or plant‐based enteral formulations available. These formulations provide macronutrients sourced from food‐based ingredients and, when consumed in the recommended volumes, provide all recommended nutrients (Table 5). It is important to note that all types of commercial enteral feedings contain food additives, including vitamins and minerals, as well as thickening and emulsifying agents.

**Table 4:** Ingredients found in standard enteral formulations.

Nutrient group	Nutrient sources
Carbohydrate	Sugars (corn syrup, sugar, or brown rice syrup,), corn maltodextrin, corn starch, fructose, isomaltulose, and corn syrup solids
Fats	Vegetable oils (canola, high‐oleic sunflower, corn, soybean, or rapeseed), medium‐chain triglycerides (coconut or palm oils), flaxseed oil, and fish oil
Protein	Whey protein isolate, partially hydrolyzed whey protein, milk protein isolate, modified milk ingredients, soy protein isolate, calcium caseinate, pea protein concentrate, and hydrolyzed sodium caseinate
Fiber	Ground soy cotyledon fiber, inulin, acacia gum, oligofructose, oat fiber, cellulose gum, pea fiber, short‐chain fructooligosaccharide, chicory root fiber, soy fiber, and soluble corn fiber

**Table 5:** Ingredients found in “real food” enteral formulations.

Nutrient group	Nutrient sources
Carbohydrate	Brown rice syrup, tomato paste, peach puree concentrate, dried green beans, cranberry juice concentrate, dried carrots, agave syrup, pea starch, kale, broccoli sprout, acai, garlic, blueberry, beet, raspberry, spinach, tart cherry, blackberry, garlic, garbanzo beans, green peas, whole‐grain brown rice, sprouted quinoa, and sweet potato
Fat	Medium‐chain triglycerides (coconut and palm kernel oils), canola oil, safflower oil, sunflower oil, flaxseed oil, and almond butter
Protein	Hydrolyzed pea protein, milk protein, dried chicken meat, and pumpkin seed protein
Fiber	Partially hydrolyzed guar gum, pea fiber, oligofructose, acacia gum, inulin, agave inulin, and locust bean gum
Other ingredients	Cinnamon, rosemary extract, coffeeberry, green tea, turmeric, dutch cocoa powder, ginger, acerola powder, and vanilla extract

### Food ingredients with emulsifying properties

Emulsifying agents have been associated with metabolic syndrome via disruption in the gut microbiome.^97^, ^98^ Maltodextrin has been shown to impair gut homeostasis via multiple mechanisms, including promoting the adherence of pathogenic bacteria to the intestinal mucus layer and inducing intestinal pathologies.^99^ Gut dysbiosis caused by some emulsifiers (eg, carboxymethylcellulose [CMC] and polysorbate 80 [P80]) is characterized by overgrowth of mucus‐degrading bacteria and decreased anti‐inflammatory cytokines.^100^ Naimi et al tested the effects of 20 commonly used dietary emulsifiers on healthy human gut microbiota in an ex vivo modeling system.^101^ In addition to CMC and P80 inducing both lasting and detrimental changes to the gut microbiota composition and function, they also found that 18 other tested agents had similar effects. All tested forms of carrageenan, guar gum, and locust bean gum significantly altered microbiome composition. Several emulsifiers significantly reduced multiple genera including both *Lactobacillus*, most driven by a decrease in *Streptococcus*, and the anti‐inflammatory *Faecalibacterium*; and several emulsifiers enriched *Bacteroides*. Interestingly, microbiota exposed to several emulsifying agents (maltodextrin, xantham gum, sorbitan monostearate, and glyceryl stearate) induced LPS, an endotoxin from the cell wall of Gram‐negative bacteria. All the tested carrageenans, as well as several gums (xanthum, guar, and locust bean) induced bioactive levels of flagellin, a component of the Gram‐positive bacteria cell wall. Together these data suggest that numerous commonly used dietary emulsifiers can disrupt gut microbiota homeostasis and enhance a microbe's ability to activate innate immune‐signaling pathways linked with intestinal inflammation.

### Food choices and diet consistency on the microbiome

A few studies have evaluated the effect of how the consumption of an exclusive oral liquid diet affects the gut microbiome in healthy participants. Johnson et al conducted a double‐blind, parallel‐arm, 17‐day longitudinal study with 34 healthy study participants randomized to receive 5% of total energy expenditure as either EVOO or medium‐chain triglyceride supplement and assessed the fecal microbiome changes before and after supplementation using shotgun metagenomic sequencing.^102^ Participants were directed to consume their habitual diet; however, two participants disclosed after the study that they consumed only a liquid meal‐replacement shake throughout the study period. Macronutrient and micronutrient profiles were relatively stable across the study period even though dietary intake regarding food choices was highly individualized. Diet accounted for 44% of the total variation in average microbiome composition. The difference in beta diversity (dissimilarity) of the fiber sources for four food groups with a known high fiber content (grains, fruits, vegetables, and legumes) was calculated. Then fiber‐source beta diversity was compared with microbiome beta diversity. This analysis showed that those who obtained their fiber from similar food sources tended to have more similar microbiome profiles. Interestingly, the extremely monotonous dietary intake of the two meal‐replacement shake drinkers supported prior findings that a less diverse diet does not induce microbiome stability; rather, diet diversity is important for the maintenance of a more stable microbiome.^103^

Tanes et al evaluated gut microbiota responses to three dietary patterns, omnivore, vegan, and synthetic fiber‐free exclusive EN (EEN), in healthy adults during three phases.^104^ The phases were 5‐day diet phase (phase 1), microbiota purge phase with antibiotics and polyethylene glycol (phase 2), and recovery phase (phase 3). The omnivore diet was designed to have a macronutrient composition similar to the EEN liquid diet except for the total lack of fiber and fatty acid ratio profiles. As expected, the purge reduced the total viable fecal bacterial load, but the EEN group had a slower recovery of the bacterial load and alpha diversity compared with the other diets. The vegan diet group was more resilient to microbiota changes caused by the purge and had the greatest diversity recovery. Compared with the vegan and omnivore diet groups, EEN induced significant changes to the microbiota composition within 3 days of the dietary phase increasing two *Rumincoccus* genera of the Clostridia cluster XIVa, (*R. gnavus* and *R. torques*) while decreasing other taxa. During the recovery phase, only in the EEN group was the proportion of Proteobacteria greater relative to Bacteroidetes and Firmicutes owing to a dominance of *Klebsiella pneumoniae* and *Enterobacter cloacae*. The gut bacteria in the EEN group also had a reduction in enzymes needed to degrade complex plant polysaccharides (fiber) and an increase in enzymes needed to digest more simple carbohydrate. These data demonstrate the importance of dietary fiber in supporting the gut microbiome composition, metabolism, and recovery following an acute ecological disruption.

In a prospective, randomized, double‐blinded, crossover study in healthy adults, Koecher et al tested the effects of 14 days of oral consumption of a fiber‐free and fiber‐containing enteral formula as well as habitual diet on the gut microbiota via FISH analysis.^105^ There was a decline in the total fecal bacteria tested during consumption of the fiber‐free formula compared with the habitual diet and fiber‐containing formula. Although the fiber intake was similar between consumption of the habitual diet and fiber‐containing formula, the number of *Bifidobacteria* and *Lactobacilli* declined during both formula consumption periods compared with the habitual diet, with the lowest numbers during fiber‐free formula consumption. The number of *Bacteroides* did not change, but there was a trend toward higher numbers of *Clostridia* on the fiber‐free compared with the habitual diet. The mean fecal pH was higher while consuming the formulas vs the habitual diet pH (7.5 vs 6.5, respectively; *P* < 0.0001) Thus, these data suggest that exclusive consumption of an enteral liquid formula for 14 days alters gut microbiome compared with the habitual diet, with a fiber‐free formula having the most dramatic effects.

Jatkowska et al.^106^ evaluated the acute effect of different doses of EN on fecal microbiota and diet‐related bacterial metabolites in healthy adults. Study participants replaced 100% (*n* = 25), 85% (*n* = 12), 50% (*n* = 12), or 20% (*n* = 12) of their daily energy requirements with a polymeric enteral formula that lacked fiber, gluten, and lactose for 7 days. Fecal samples collected at baseline and day 7 revealed all EN groups, except 20% EN, had a shift in gut microbiota structure in a dose‐dependent manner as assessed by 16S rRNA sequencing. The 100% and 85% EN groups showed the most significant changes in taxon‐relative abundance, with fewer changes observed in the 50% and 20% groups. At phylum level, 100% EN decreased Bacteroidetes abundance and increased *Desulfobacterota* levels, 85% EN increased Proteobacteria abundance, and 50% EN decreased Actinobacteria and Bacteroidetes abundances. The lack of fiber in 100% EN coincided with decreases in fiber‐fermenting and SCFA‐producing taxa and SCFAs (acetate, propionate, and butyrate) and increases in potentially harmful organisms. Many of these changes overlapped with the 85% EN group. All EN groups had an increase in fecal pH. Interestingly, although gut microbiota shifts were noted in the 50% EN group, adherence to a diet pattern for the remaining 50% of energy needs consisted of high intakes of fish/fish dishes, vegetables, potatoes, nonalcoholic beverages, and low consumption of cereal/cereal products. Milk/milk products and meat/meat products were negatively correlated with these microbiota changes. These data show that EN modifies the gut microbiome in a dose‐dependent manner and that dietary fiber should be provided when it is not contraindicated to promote gut microbiome composition and function.

### “Real‐food” enteral ingredients vs ready‐to‐feed standard enteral ingredients

With the increased knowledge of the benefits of eating a variety of whole foods on health, including gut health, there has been a growing consumer demand for real‐food enteral feeding products owing to concerns about the processed ingredients and additives in standard enteral formulations.^107^ Blenderized tube feedings (BTFs) have been shown to improve GI symptoms including reflux, abdominal pain, diarrhea, and quality of life.^108^, ^109^ Presented here are the limited available studies evaluating the effects of enteral BTF formulations on the gut microbiome.

Gallagher et al recently evaluated the feasibility of using homemade BTFs in a complex pediatric population, and, in addition to assessing clinical outcomes, they also evaluated the gut microbiome.^109^ Twenty pediatric (mean age 3.4 ± 2.2 years) outpatients fed via a gastrostomy tube were transitioned from a commercial enteral formula to a BTF formula over 4 weeks and were monitored for 6 months. Participants required 50% more energy with the BTF compared with the commercial formula to maintain their BMI. Bacterial diversity (Shannon index) and richness (Chao1 index) increased, and Proteobacteria decreased with the BTF.

Katagiri et al conducted an observational study in 11 pediatric patients receiving either homemade BTFs (*n* = 5) or a ready‐made commercial tube feeding formula (*n* = 6) via gastrostomy (*n* = 10) or enterostomy (*n* = 1) tubes evaluating the effects of formula on the oral and gut microbiome.^110^ Oral microbiota composition differed slightly but significantly between groups (*P* < 0.041), and the gut microbiota composition differed significantly (*P* < 0.0017). Only the gut microbiome alpha diversity increased significantly with the BTF. The relative abundance of the phylum Proteobacteria, class *Gammaproteobacteria*, and genera *Escherichia‐Shigella* were significantly lower, and the genus *Ruminococcus* was increased in the BTF group. Although the relative abundance of microbial composition did not differ between groups in the oral microbiota, 137 functional profiles exhibited differences between groups, notably sulfur and methane metabolism, and carbon‐fixation pathways in prokaryotes were enriched in the BTF group. The gut microbiome showed 271 gene metabolic functional profiles in the BTF; notably, carbon‐fixation pathways in prokaryotes were enriched.

## CLINICAL CONSIDERATIONS

- The gut microbiome supports intestinal and overall health, and diet is one of the main factors driving its composition and function.
- It is important to know what foods people are eating in the context of their clinical condition and health.
- The food sources of macronutrients and micronutrients impact the gut microbiome.
- Fiber is an important dietary component, aiding with digestion, bowel motility, and lowering cholesterol, yet most people consume less than half of what is recommended daily.
- Dietary fiber from a variety of food sources is essential in supporting gut microbiome composition, function, and resilience to change.
- Enteral formulations typically provided to patients are ultraprocessed containing many synthetic ingredients. These formulations also often lack fiber, especially for formulations fed to critically ill patients, which is concerning as during critical illness the gut microbiome composition and function are disrupted.
- Fiber is a component of many foods (eg, fruits, vegetables, and legumes), and these foods also contain a variety of phytochemicals that support a healthy gut microbiome composition and function. Therefore, consuming a varied diet with whole foods is important for supporting a diverse and stable gut microbiome.
- Early studies with real‐food formulations in nonhospitalized patients are suggestive as being superior in supporting the gut microbiome compared with standard enteral formulations.
- The gut microbiome changes throughout the life cycle. Most chronic diseases are linked with an altered microbiome. These considerations along with the fact that most patients may not be consuming adequate fiber should be included in the nutrition care plan.
- Fiber is often deemed the culprit for causing diarrhea in enterally tube‐fed patients, so gradually introducing fiber to patients is important to allow the microbiome to adjust accordingly.

## SUMMARY AND FUTURE DIRECTIONS

Over the past decade, we have come to realize the importance of the gut microbiome in maintaining optimal digestion, producing key biological metabolites, and supporting gut integrity and immunity. We have learned that dietary input is a key factor in influencing gut microbiome composition, diversity, and function. (Table 6) Primarily with association studies, links between the gut microbiome and many chronic diseases have been recognized. However, whether the gut microbiome is driving disease, disease is driving gut microbiome, or it is a combination of both processes is not well understood. Most available human data are derived from healthy participants, and there is a gap in knowledge as to how different food inputs impact the microbiome of individuals with chronic diseases or those requiring EN support. As we become more cognizant of how food consumption impacts not only our health but also our gut microbiome, we should be considering providing real foods to patients dependent on enteral feeding. The initial studies that have been done evaluating the effects of real‐food–based enteral formulas on gut microbiome in nonhospitalized patients are suggestive that they are well‐tolerated and improve gut microbiome composition and function. However, more research is needed into how real‐food–based enteral formulas impact adults, particularly adults in acute care settings. Future research consisting of well‐designed rigorous studies in patients requiring enteral feedings focusing on the assessment of these formulations for their safety, impact on the gut microbiome, and patient clinical outcomes is warranted.

**Table 6:** Summary of dietary factors on gut microbiome.

Food component		Gut microbiome diversity	Other impacts
High‐fat vs low‐fat diet		Decreased alpha diversity	High‐fat diet decreased SCFA and increased proinflammatory metabolites
Plant‐based vs omnivore diet		Increased beta diversity	Omnivore diet decreased carbohydrate fermentation metabolites and increased amino acid fermentation metabolites
Dietary carbohydrate	Dietary fiber	Increased alpha diversity	Increase SCFA levels
	Fruits and vegetables	Increased alpha diversity	Increase SCFA levels
	Simple sugars	Decreased alpha diversity	–
Dietary fat	Avocados	Increased alpha diversity	Decreased blood inflammatory cytokines
	Olive oil	Increased diversity	Several taxa correlated with markers of metabolic syndrome
	Fish oil vs lard or soybean oil	Fish oil increased Proteobacteria phylum and genus *Desulfiovibrio*	Fish oil increased inflammatory gene expression in the colon
	Saturated vs unsaturated fats	Decreased alpha diversity	–
Dietary protein	Nonmeat vs meat‐based	Increased beta diversity	–
	Plant‐based vs animal‐based	Increased beta diversity	Higher SCFAs, lower gut pH, and lower ammonia concentration
	Soy protein vs casein	Increased alpha diversity	Increased SCFAs
	Pea protein	Increased alpha diversity	Increased SCFAs

Abbreviation: SCFA, short‐chain fatty acid.

## AUTHOR CONTRIBUTIONS

Gail A. M. Cresci solely contributed to the conception and design of the research, interpretation of the data, writing of the manuscript, and approval of the final manuscript.

## CONFLICT OF INTEREST STATEMENT

Gail A. M. Cresci has served as a speaker in the past 5 years for Nestlé Health Science and Kate Farms Medical.
